# The relevance of science-policy-practice dialogue. Exploring the urban climate resilience governance in Italy

**DOI:** 10.1186/s40410-021-00137-y

**Published:** 2021-07-01

**Authors:** Ombretta Caldarice, Nicola Tollin, Maria Pizzorni

**Affiliations:** 1 Interuniversity Department of Regional and Urban Studies and Planning, Politecnico di Torino, Responsible Risk Resilience Centre, viale Mattioli 39, 10125 Torino, Italy; 2 Civil and Architectural Engineering, University of Southern Denmark, Odense, Denmark

**Keywords:** Urban climate resilience, Local climate action, Multiscalar governance, CitiesIPCC, Science-policy-practice dialogue

## Abstract

The concept of resilience has been developed for over 40 years in different disciplines. The academic discussion on defining resilience is thriving to create interdisciplinary understandings and meanings. Simultaneously, resilience has firmly entered into planning practice to address vulnerabilities and cities' exposure facing to present and future hazards particularly related to climate change effects. In the last twenty years, a growing number of cities are adopting local climate actions, and urban resilience is also gradually a crucial part of international and national policies worldwide. Despite the increasing attention to urban resilience, its implementation at the local scale and the required increasing ambition are still lagging, also due to a lack of dialogue among researchers (the scientific level), policy-makers (the normative level) and practitioners (the operational level). Following the 2018 CitiesIPCC Research and Action Agenda recommendations, this paper contributes to improving understanding barriers, opportunities, and needs for science-policy-practice dialogue for urban climate resilience. The paper analyses the urban climate resilient strategiesstrategies of the Italian metropolitan cities, concluding that a science-policy-practice dialogue is lacking in implementing evidence-based climate change resilience policies and actions actions at the local scale. Starting from the Italian case study, the paper suggests an iterative process to unlock the science-policy-practice dialogue for contributing to operationalise urban climate resilience fostering thanks to a multiscalar governance approach.

## Introduction. The rise of resilience in science, policy and practice

The 2018 Revision of World Urbanization Prospects by United Nations powerfully highlights the growing speed of urbanisation worldwide, implementing the progressive and uncontrolled “*planetary urbanisation*” (Brenner and Schmid 2014). Today, 55% of the world population lives in urban areas (73% in the EU with a rising trajectory that will reach 82% in 2050). Consequently, cities account for 60–80% of global energy consumption and the same share of CO_2_, producing 50% of global waste, consuming 75% of natural resources and generating 80% of global GDP. Cities face critical global challenges, mainly related to environmental changes, social crises, critical infrastructure failures, terrorist attacks, technological accidents, and the recent pandemic.

The 2018 CitiesIPCC Research and Action Agenda outlines that cities are “*open, complex, self-organising, adaptive, and evolving formations that are embedded in broader social, ecological, economic, technical, institutional, and governance structures*” (Prieur-Richard et al. 2018). As a complex environment, urban areas and their regional systems dually represent the most significant threat (e.g., negative externalities and consumption) and the most relevant opportunity (e.g., innovation and creativity) for sustainable development due to the concentration of human activities (Brunetta et al. 2019). The strategic importance of cities is evident in the face of global challenges as climate change, natural resource management, sustainable production and consumption, and community development. The traditional emphasis posed to the city as an accelerator of the economic system (Jacobs 1970), an engine of globalisation (Sassen 2013), and an enabler of innovation (Concilio and Tosoni 2019; Glaeser 2011), today leaves the stage to the emerging awareness of the importance that cities mean for the global environmental change and social transition (Seto et al. 2013), and about the interrelation between climate emergencies and urban socio-economic opportunities (Coalition for Urban Transitions 2019). Cities can and shall be significant catalysts of change in implementing actions for climate adaptation and mitigation in the face of multiple concurrent crises.

To provide a broad answer to climate change and socio-economic uncertainties, cities worldwide have already started to develop specific mitigation and/or adaptation policies/plans/actions in the perspective of urban climate resilience^1^. However, relatively few efforts have been done regarding the development of policies, plans, and initiatives concerning adaptation instead of mitigation, because it is easier to quantify and evaluate emission reduction solutions than the effectiveness of adaptation actions (Carmin et al. 2012; Harman et al. 2015). When researchers, policy makers and practitioners focus on adaptation, they mainly concentrate on the study, evaluation, and planning of risk assessment in specific sectors, such as water management, energy and infrastructures (Carmin et al. 2012). Consequently, investigating how policy agendas can be implemented in cities is poorly and it has low influence on planning and practices (Harman et al. 2015). Nevertheless, a growing number of cities are now pioneering an urban resilience approach, facing challenges such as the uncertainty and the unpredictability of the phenomena they are addressing, although suffering for lack of knowledge in terms of data, evaluation methods/tools, planning skills, and lack of institutional, financial and technological capacities.

To support cities’ initiatives, many international organisations and policy circles promote resilience so that the concept has emerged as a central aspect. In this perspective, the SDG 11—Make cities and human settlements inclusive, safe, resilient and sustainable aims to support cities in adopting adaptation, mitigation, resilience, and disaster relief plans (UN-Habitat 2020). Moreover, the Article 4 of the Paris Agreement, in his Article 4, states that “*eEach Party shall prepare, communicate and maintain successive nationally determined contributions that it intends to achieve. Parties shall pursue domestic mitigation measures, with the aim of achieving the objectives of such contributions*” (UN 2015). Finally, the New Urban Agenda commits to promote international, national and local action including adaptation and mitigation of climate change, supporting resilience building and the reduction of greenhouse gas emissions from all relevant sectors, in a manner consistent with the Paris Agreement (NUA 2016). In a nutshell, resilience has become a topic for academic researchers, policy-makers, and practitioners (Haupt and Coppola 2019).

However, what we speak when we speak about urban resilience? The terms resilience is derived from the Latin verb “resilio” which means to leap back, spring back, rebound, shrink, retreat, and give up. It is a composite word formed by the prefix “re-”, usually defining the reiteration of action and sometimes used to reiterate a concept, and the verb “salio” meaning to jump, to bounce, but also to flow (Folke 2016). The resilience concept has no unique meaning. There is not a broad consensus on its usage as the term is used in different disciplines and context with much-differentiated understanding and references (Moser et al. 2019). The common ground of all the different meanings of resilience is that local and global dynamics and events—which nature is significantly diversified—threaten our societies and our cities. Whilst there is a large number of interpretations about principles and characteristics given to resilience from science, policy and practice, in this paper we assume that resilience aims at increasing the ability of urban systems to respond systemically and dynamically to present and future shocks related to significant global challenges as unsustainable development patterns, rapid and unplanned urbanisation, climate change, and social inequalities (Brunetta and Caldarice 2020).

Following this more profound meaning, defined “co-evolutionary resilience” (Davoudi 2012), resilience is not simply the opposite of vulnerability (White and O’Hare 2014) but a broad concept whose final scope is to prevent and manage unforeseen events together with the improvement of the environmental and social quality of an urban system (Meerow et al. 2016). In the science dimension, the term resilience is used to deal with environmental (therefore climate) issues and social issues. In this perspective, resilience should be built at various levels and in a cross-sectoral manner, including assistance programmes for vulnerable communities, prevention projects (such as warning systems and disaster insurance) and integrating climate change adaptation and risk management into development programmes (Romero Lankao and Qin 2011; EU 2013). The importance of the social aspectdimension of resilience was also emphasised in the Recovery and Resilience Facility (RRF) entered into force on 19 February 2021. Within the programme, the EU aims to support reforms and investments undertaken by the Member States to mitigate the economic and social impact of the Coronavirus pandemic and make European economies and societies more sustainable, resilient and better prepared for the challenges and opportunities of the green and digital transitions (EU 2021). Hence, resilience motivates the transformative potentials of cities (DeVerteuil and Golubchikov 2016). It becomes instrumental in addressing both causes and effects of these significant global challenges, rethinking how cities are designed, planned, and managed while fostering innovation.

Although the theoretical debate on urban resilience is deeply investigated, several methodological challenges remain mainly related to the concept’s practical sphere (Crowe et al. 2016). The key challenge for urban resilience is to co-develop and harmonise scientific and practice-led knowledge to support informed and science-based decision and policymaking to enable our cities to evolve and innovate. Thereby, urban resilience implementation is the central policy challenge for the 21st-century research agenda (Pitidis et al. 2018).

From a planning perspective, urban resilience requires to rethink the planning process's rationality radically, as not any longer aimed at the realization of physical infrastructures but at reducing environmental emergencies and social fragilities of cities. This premise opens up a new season for spatial planning, which requires new spheres of action that deal with complexity (Rauws 2017). If spatial planning has mainly oriented to regulate anthropic transformations of the environment, the contemporary dynamics call for new planning tools taught to urban resilience. This new approach, which proactively faces urban risks and reduces human impact on the ecosystem, would guide cities towards new development trajectories in a co-evolutionary perspective sealing urban resilience (Eraydin and Taşan-Kok 2013). Operatively, urban resilience requires to successful improve “mainstreaming adaptation” into local climate action and regulatory frameworks. It can help planners rethink traditional approaches to land use and infrastructure design based on past trends, move toward more forward-looking risk-based design for a range of future climate environmental conditions, and reduce administrative cost by strengthening resilience through existing policy channels (Uittenbroek et al. 2013). On the opposite side, resilience is mainly used to local climate action, setting tight boundaries on his action from a social perspective. Following this direction, resilience embraces climate change adaptation, mitigation actions, and disaster risk reduction while recognizing the complexity of rapidly growing urban areas and the uncertainty associated with climate change.

As evidence above, the framework of reference about urban climate change resilience is certainly complex, and can be confusing at times.

From this viewpoint, the paper intends to go beyond the mere description of the characters and features of urban resilience, aiming at contributing to the current debate on the operationalisation of urban climate change resilience. The first section explores the relevance of science, policy and practice dialogue in enabling urban climate change resilience. In the second section, the Italian approach to the operationalisation of urban climate change resilience is presented. The third section discusses the main gaps in the current Italian approach to urban climate change resilience implementation. The conclusion highlights the main enabler factors to operationalise urban climate change resilience. Besides, an iterative process to unlock the science-policy-practice dialogue broadly applicable in implementing and operationalising urban climate change resilience fostering a multiscalar governance approach is provided.

## Background. Urban resilience from theory to practice

The concept of urban resilience has emerged as a call to reframe spatial planning theory and practice in the face of environmental, social and economic vulnerabilities of cities linked to the sustainability science (Curtin and Parker 2014; Elmqvist et al. 2019). Cities worldwide are gradually developing climate mitigation and adaptation strategies. Reckien et al. (2018) outlined that 66% of EU cities have a mitigation or adaptation plan in place. The top countries were Poland—where 97% of cities have mitigation plans—Germany (81%), Ireland (80%), Finland (78%), and Sweden (77%).

To describe the pathway towards urban resilience’s operationalisation, the paper considers the planning system as a technical core embedded in an institutional framework (Servillo and Van den Broeck 2012). This paper supports that institutional frame is essential in applying resilience within a planning system to highlight dominant norms, perceptions and paradigms which can lead to urban resilience actions (or lack thereof) in the face of crisis. In this perspective, public institutions from national to local are not considered as given or static, but as social products that are actively created, adapted and maintained as coherent through action, to meet the never-ending changes in society (Caldarice and Cozzolino 2018). Moreover, this new institutional understanding of urban resilience opens up promising research perspectives about urban change (Lang 2011). In other words, the rapid political ascent of urban resilience raises important questions around how the concept is understood, what it is designed to achieve, and how this may translate into practice (Doyle 2016). Nadin et al. (2020) outlined that climate change, energy security and social injustice are the emerging witched problems of contemporary cities that allow transferring the government’s ability and capacity to deliver integrated, adaptive and collective planning decisions.

A deeper understanding of the practical realities around applying and implementing urban resilience strategies at the local level highlights some critical knowledge gaps. Based on Caldarice et al. (2019), we can identify three challenges:

1. Systemic challenge: how to decipher and integrate the different and conflicting understandings and interpretations of urban resilience transition, to communicate and effectively support informed decision-making;
2. Policy challenges: how to strength multi-level governance for urban resilience favouring collaboration and harmonisation of policies and actions across national and local governments; and
3. Co-Benefit challenges: how to define and reinforce the co-benefits potentially produced by urban resilience transition, integrating mitigation and adaptation actions.

These cross-cutting knowledge gaps underline how we need to facilitate the discussion on how to strength multiscalar governance in the frame of supporting transformative climate change responses inspiring the next frontier of research focused on the science of cities and climate change.

We can argue that these challenges arise mainly from the lack of interface and disconnect among science, policy and practice in implementing urban climate change resilience. The science-policy-practice dialogue is a catchall term to describe the processes and settings in which decision-makers deal with scientific research in orienting their thinking, analyses or decision-making to be applied by practitioners (van den Hove 2007). As outlined in the 2018 CitiesIPCC Research and Action Agenda (Prieur-Richard et al. 2018) and by the ICLEI 2018 Resilient Cities Report ), catalyse collaboration and knowledge production among researchers, urban practitioners and policy-makers are crucial to have a constructive, open, long-term and iterative dialogue to match current and future knowledge needs, to respond to challenges faced by cities. Building from the knowledge shared by the science, practice and policy communities will be essential to support the urban resilience implementation.

If theoretically, the relevance of the interaction among science, policy and practice is clear, which are the main barriers to activate it? At first glance, researchers, policy-makers and urban practitioners operate at different time and spatial scales, with different agendas, and using different languages and narratives. At the same time, they pursue the same objective: support cities to take actions to increase ambition to climate targets and take transformational action against multiple crises. In Table 1, some weak elements of the science-policy-practice dialogue are underlined.

**Table 1:** Key topical knowledge gaps in science-policy-practice dialogue

	Knowledge gap	Description
Research	How to find the right ‘fit-for-purpose’ definition of resilience?	Resilience is characterised by epistemological agility to serve as a useful lens for policy narratives in dealing with different contexts and problems. Seldom this caused a broad definition of resilience so that it has been almost meaningless. The most challenging point is how to frame urban resilience as a powerful agent and a driver of change for cities in transition
Policy	How to strength the resilience policy coherence at the local scale?	Cities need to work to align their actions and visions in the context of pursuing the international policies that pursue resilience. The most challenging point is to strength multiscalar governance for urban resilience favouring collaboration and harmonization of policies and actions across national and local governments
Practice	How to support the resilience in urban projects?	Resilience needs to develop frameworks and tools that enable the integration of climate considerations into fiscal and financial decision-making at the city scale. The most challenging point is how to include low-income and other marginalised urban inhabitants in fiscal and financial decision-making

Source: Authors’ elaboration based on Prieur-Richard et al. 2018

Recently, the 2020 JRC Time For Transformative Resilience Report defined resilience in a transformative dimension. Within this approach, the crisis is an “*opportunity to progress and bounce forward through a combination of adaptation and transformation measures*” (Giovannini et al. 2020). According to this definition, it is possible to state that the co-evolutive resilience, theoretically defined by Davoudi (2012), is today applied as transformative within policies (as experimented in COVID-19 emergency). In this perspective, it is interesting to highlight the difference in terminology between science, policy and practice: resilience is defined “co-evolutionary resilience” by academics (Brunetta and Caldarice 2020), “transformative resilience” by policy-makers (Giovannini et al. 2020), and finally merely “resilience” by practitioners as commonly a synonymous of sustainability in practice (Meerow and Stults 2016).

## Case description. The Italian way to operationalize urban climate resilience

Italy is one of the European countries most affected by climatic and environmental risk and hazards, particularly hydrogeologic ones. Based on the International Disaster Database (https://www.emdat.be), there have been more than 140 events from 1900 to date, causing the highest number of human losses and the most significant economic damage in a European context (reportedly almost one billion dollars). More on this point, the 2020 Italian NGO Legambiente Report on cities and climate change in Italy (Legambiente 2020) confirms the growing trend of violent disasters, highlighting that between 2010 and 2020 Italy suffered extreme weather events affecting 507 municipalities. Despite this situation and the widespread awareness of climate change as an emergency, the Italian pathway to adaptation is still at an initial phase (EU 2018). Additionally, as outlined by Reckien et al. (2018), Italian legislation does not require local governments to develop local climate plans towards mitigation and/or adaptation—differently from Slovakia (compulsory local mitigation plans), Denmark (binding local adaptation plans), France and UK (in which both local mitigation and adaptation plans are mandatory).

About the mitigation target, the Italian Ministry for the Environment, Land, and Sea (IMELS), in charge of climate change policy, supported the development of the 2002 National Action Plan to reduce GHGs and implemented some of its contents in 2013 following the 1997 Kyoto Protocol. In 2020, IMELS released the National Energy and Climate Plan (*Piano Nazionale Energia e Clima* 2030—PNIEC), aiming to strongly reduce the GHG emissions and fossil fuels energies and related consumptions. Simultaneously, the Italian Ministry of Economic Development (IMED) released in 2017 the Italian National Energy Strategy (SEN), which aims to make the national energy system more competitive, sustainable, and secure. The SEN means to align Italian energy prices with the European ones to: (i) benefit for both companies and consumers; (ii) to contribute to decarbonisation in line with the long-term targets of the Paris Agreement on Climate Change; (iii) to improve energy efficiency encouraging energy conservation to mitigate environmental and climate impacts; (iv) to promote environmentally conscious lifestyles from sustainable mobility to wise energy usage; and (v) to improve the security of energy supply, and to strengthen Italy’s energy independence.

About the adaptation target, IMELS has approved in 2015 the Italian National Adaptation Strategy to Climate Change (NAS). NAS is a tool for encouraging cross-sectoral adaptation actions in planning activities at the national, regional and local level. IMELS has been working on the implementation of the NAS through the development of the Italian National Adaptation Plan for Climate Change (NAP) which will provide a set of adaptation actions in order to adapt and take advantage from climate change for the most vulnerable sectors already identified within the NAS (e.g., water resources, soil degradation, hydrogeological risk, biodiversity and ecosystems, health, agriculture, forestry, energy, tourism, and urban settlements). The NAP, currently in the strategic evaluation assessment phase, represents the national strategic framework for other ministries, regions, local authorities related to the integration of adaptation within policy processes.

**Fig. 1:**
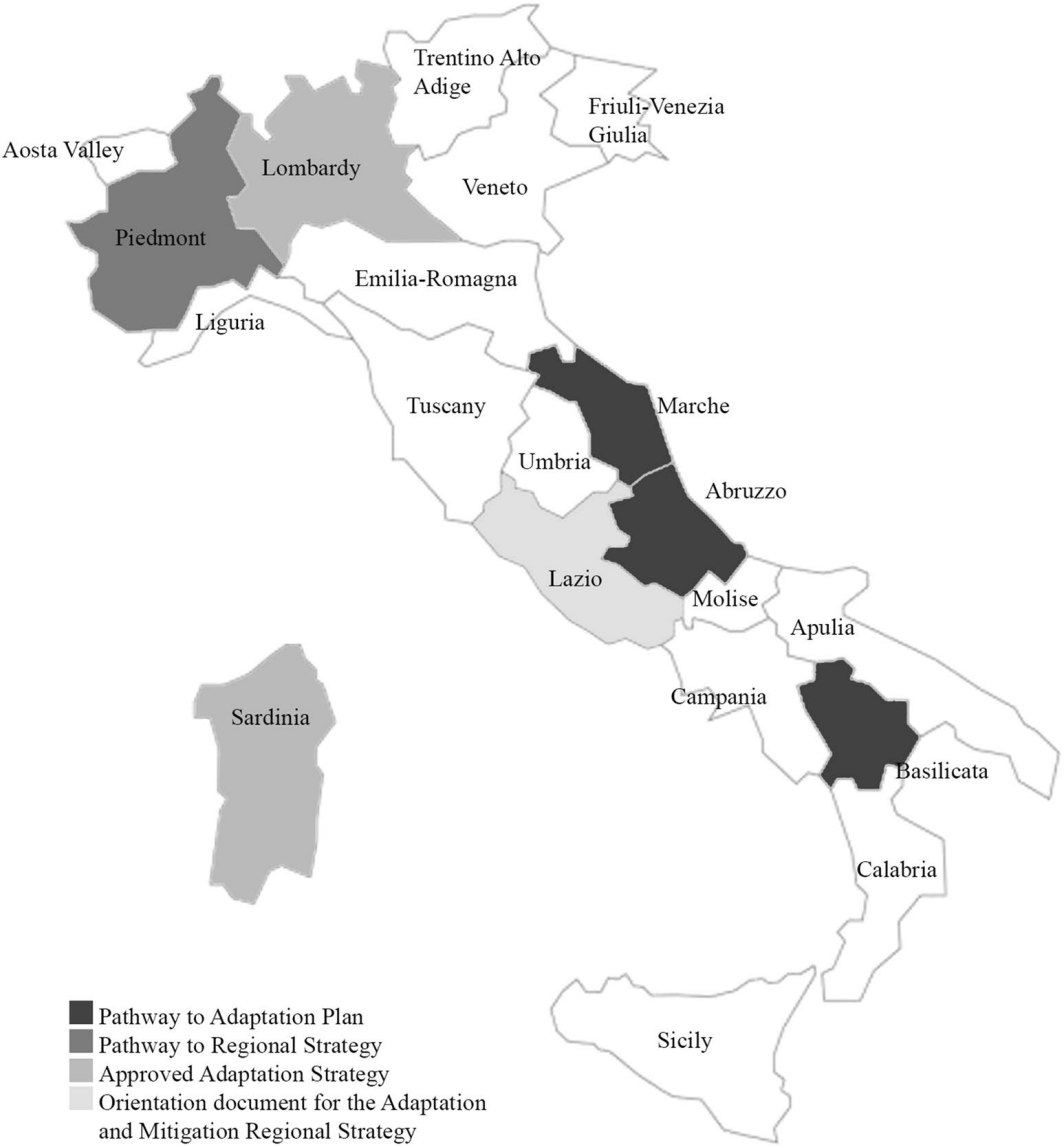
Overview of regional resilience initiatives (updated 2020). Source: Authors’ elaboration

At the regional level (Fig. 1), Lombardy (2014 | 2016), Emilia-Romagna (2018) and Sardinia (2019) are pioneering regions developing a regional strategy and/or plan to mitigate and/or adapt to climate change. The other regions (mainly the southern ones) are little involved in these strategic processes despite the strong evidence pointed out by the IPCC and EEA about the Mediterranean basin’s increasing vulnerability to climate change. Furthermore, Lazio has even launched the preparation of the regional action strategy, and Abruzzo, Marche and Basilicata have launched the regional plans that are now producing a comprehensive climate analysis and outlining the possible way of actions. Lastly, Piedmont has approved the orientation document to develop the regional adaptation and mitigation strategy (2020).

At the local level (Tables 2 and 3), the fourteen Italian core cities at the metropolitan level (e.g., Rome, Milan, Naples, Turin, Palermo, Genova, Bologna, Florence, Bari, Catania, Venice, Messina, Reggio di Calabria, and Cagliari) are working both on mitigation and adaptation in different ways. In line with the European Covenant of Mayors Initiative (2008), thirteen of fourteen cities approved their Sustainable Energy Action Plan (SEAP) from 2010 to 2018 (Reggio di Calabria is the only missing one), formalizing the Mayors institutional commitment to reduce GHG emissions on their cities and to improve energy saving and efficiency at both urban and building scale. Apart from the stand-alone approach of SEAPs, mitigation perspectives are often mainstreamed in several other planning documents, as land-use plans (usually noticed in the case of current plans adopted no later than ten years ago), green regulation (with a focus on the green provision, air quality benefits, plantation, carbon storage, and soil and biodiversity protection), and building regulation (where energy saving and efficiency issues become central for buildings and public space). In contrast with the widespread of SEAPs, adaptation orientations are less numerous: Bologna is the only metropolitan city with an adaptation plan (Bologna Local Urban Environment Adaptation Plan for a Resilient City approved in 2015) that aims to make the city more resilient and able to meet the climate change challenge, Rome has recently approved a Resilient Strategy in the frame of 100 RC, while Venice has partial and ongoing commitment related to the C40 Network—2020 Deadline for draft an action plan for the climate. European projects also cover a remarkable role in adaptation, with several cities are participating in LIFE and HORIZON 2020 call for projects.

**Table 2:** Overview of the core metropolitan cities resilience initiatives (update 2020).

Core metropolitan city	Inhabitants (2017)	Metropolitan cities initiatives		
		Mitigation	Adaptation	Networks
Turin	882.523	SEAP (2010)	Climate Resilience Plan (2020)	–
Milan	1.366.180	SEAP (2018)	Land use plan (2019)	100 RC C40
Genova	580.097	SEAP (2010)	On-going	–
Venice	261.321	SEAP (2012) on-going new SEAP (2022)	On-going	C40
Bologna	389.261	SEAP (2012)	Adaptation Plan (2015)	-
Florence	380.948	SEAP (2011)	on-going	
Rome	2.872.800	SEAP (2013)	Urban Resilience Strategy (2018)	100 RC C40
Naples	966.144	SEAP (2012)	–	–
Bari	323.370	SEAP (2011)	–	–
Reggio di Calabria	181.447	–	–	–
Palermo	668.405	SEAP (2013)	–	–
Catania	311.620	SEAP (2015)	on-going	–
Messina	234.293	SEAP (2015)	–	–
Cagliari	154.106	SEAP (2014)	On-going	–

Source: Authors’ elaboration

**Table 3:** Description of the Italian planning system

Level	Spatial Planning Laws	Spatial Planning Tools	Urban Resilience Tools
National	National planning law	–	National Adaptation Strategy (NAS) National Adaptation Plan (NAP)
Regional	Regional planning laws	Regional landscape plan	Not compulsory Regional Strategy or Regional Action Plan
Provincial or Metropolitan	Provinces and Metropolitan Cities do not have legislative power	Province Territorial Plan Metropolitan Strategic Plan	There is no a specific commitment for strategy or plan. Metropolitan Strategic Plan can insert some resilience orientations
Local	Land-use plans legally binding	Land-use plan	Local Mitigation Plan (subscribing Covenant of Majors) Not compulsory Local Adaptation Plan

Source: Authors’ elaboration

Three cities are part of international initiatives mainly related to city-networks working on urban resilience: Rome and Milan are members of the 100 Resilient Cities, while Venice is a C40 Member City. In particular, Rome released in June 2018 the Resilient Strategy built in order to create strong synergies with other strategic urban resilience programs and initiatives currently being implemented in the city, such as the Smart Mature Resilience Project financed under the Horizon 2020 program, and the Resilient Urban Agriculture and Landscape Project under the Urbact European program. Additionally, the Rome Resilient Strategy is compliance with the Fossil Fuel Free Streets Declaration of C40 and the Sustainable Energy and Action Plan (2013), so that Rome undertook to have a zero-emission city area by 2030. Rome Resilient Strategy is based on four pillars: (i) an efficient city at the service of citizens; (ii) a dynamic, strong and unique city; (iii) an open, inclusive and supportive city; and (iv) a city that protects and enhances its natural resources. Next step of implementation for the city will be to define public and private funding to implement actions that make Rome stronger and more resilient. Differently, Milan decided to set a Chief Resilience Office (2017) thanks to the Rockefeller Foundation support and decided to insert strategic objectives and measurable indicators of urban resilience in the currently approved land-use plan (2019). In particular, urban resilience will play a significant role in the implementation of the Objective 3—“A green, liveable and resilient city” that will include top initiatives related to restoring and consolidating a series of underutilised public and private spaces into ecological corridors, as well as incentivising the environmental sustainability of existing and new construction through the introduction of new buildings standards.

This brief analysis of the Italian approach to urban resilience’s operationalisation points out that the Covenant of Mayors widely enhanced mitigation plans among major Italian cities (and smaller ones) in the last decade. It does not happen the same for adaptation as the Italian National Adaptation Strategy to Climate Change (2015) is not prescribed that adaptation plans have to be compulsory for cities. Differently from mitigation, in some few cases, the adaptation approach is mainstreamed into official documents. However, this scenario reveals a fragmented situation for Italian cities that still need central support and coordination to systematically undertake their climate commitment (Pietrapertosa et al. 2019). In line with that, both national and regional initiatives and land-use plans should become a more robust benchmark for cities, providing the necessary contributions in political, technical and financial resources. More explicitly, both national policies and supportive policies by the leading international organisations are missing to mainstream a new course in the global resilience effort to support cities to take their responsibilities and actions on the international stage.

## Up for discussion. Highlighting Italian gaps in urban climate resilience

Since in this paper we assume that resilience is embedded within an institutional perspective, it means resilience has to be framed in a juridical organisation connected to planning systems. Janin Rivolin (2017) outlined that the Italian planning system is characterised by a conformative approach, distinguished by binding zoning. Urban transformation strategy is inserted into a land-use plan that assigned land use and transformation rights preventively. As outlined in , the Italian planning system is strictly hierarchical, and it is structured at four-levels: a national strategy, a regional law, a territorially coordinated plan at province scale (or metropolitan scale for 14 metropolitan areas), and a municipal land-use plan detailed thanks to implementation plans.

The Italian planning system structure allows us to underline three weak elements in operationalising urban resilience.

Firstly, the Italian institutional frame analysis shows a lack of integration between planning and environment, rooted in the Italian legislative approach. The Reform of Title V of the Italian Constitution (2001) sought to solve the conflict between environmental and planning responsibilities shared between State and Regions. Currently, environmental protection is entrusted to the State, while spatial planning is a State-Region matter. On the climate side, the National Adaptation Strategy (NAS) should be the tool that regulates the subordinate planning levels, while the environmental (and regional) scale should identify strategies for climate change. More on this point, Italian policies and strategies seem to work on the environmental side of resilience (therefore climate). What often gets missed is the social dimensions of resilience. Urban climate resilience policies and strategies continually focus on a “deficit concept”, like vulnerability, rather than studying the strengths of communities and societies in facing effects of climate change.

Secondly, climate adaptation and mitigation are not legally binding. Regional tools define strategies according to national guidelines, but where national guidelines do not exist, they act autonomously. However, on the local scale, public authority assigns rights of use and development of the land through a binding local plan. For these reasons in Italy, it is fundamental to leverage the local level to make planning operational, build climate change capacity, create the financing, and exchange knowledge between local institutions, and then also through vertical subsidiarity.

Thirdly, Italy manages adaptation in the perspective of urban climate resilience in two ways: the first is a mainstreamed adaptation approach, integrating adaptation into local plans, as happens in Milan and Bologna; the second is a sectoral adaptation approach, which provides specific sector plans to integrate adaptation into planning, as happens in Turin and Rome. This second type of improving adaptation to climate change, the sectoral one, is the most problematic because Italy is the only European country still working on sectoral and separated plans. Furthermore, urban resilience in Italy is declined exclusively as the adaptation to climate change, excluding economic and social issues and giving priority to ecology. This dynamic is more accentuated in sectorial planning, allocating adaptation to climate change to the environmental sector (such as Turin). We should consider that resilience is an ecological issue and an urban issue, which should include structural changes, such as environmental, economic, and social challenges. In a nutshell, while the local level is the only legally binding, cities do not need to develop a local climate plan compulsory.

At the end of thise overview, we support that the critical interaction among science, policy and practice can unlock the enhancement options of the Italian policies and strategies for urban climate resilience. We propose some lessons for improving the governance and building capacities of climate change resilience that can be learnt from the Italian case study presented in this paper. For sure, better vertical integration and mainstreaming among policies and practices could be supported by a satisficing sharing of information, experience, knowledge bases and good practices suitable to be transferred across different territories. Together with this aspect, sciencethe research can support urban climate resilience processes showing operative replies supporting the lack of professional experience with adaptation planning for administrations at the sub-national and sub-regional levels, and showing guidelines and methodologies developed for planning at a specific level suitable to be applied to different territorial scales or locations.

## Conclusion. Looking for a fruitful dialogue between science, policy and practice

As stated in the introduction, understanding how to improve the relationships between science, policy and practice has been described as one of the critical challenges for sustainable development in the twenty-first century.

We demonstrate that the science, policy and practice interface have to be stress out to co-design, co-produce and share knowledge and information to empower cities to take more ambitious climate action. Research into the boundaries of the science–policy interface enables a deeper understanding of how to manage the challenges around communication and collaboration that arise from science–policy interactions.

To solve the critical limiting factors, i.e., financial, institutional and technological capacity issues, urban resilience needs to work at different scales, with different times and terms between researchers, policy-makers and practitioners. More on this point Prieur-Richard et al. (2018: 14) stated that “*as researchers, urban practitioners and policy-makers often operate at different time and spatial scales, and use different vocabularies, it is important to distil the information already available to meet the immediate knowledge needs of cities, and to have a constructive, open, long-term and iterative dialogue to match current and future knowledge needs, to respond to challenges faced by cities*”. This dialogue for progress in addressing climate change must be built withstanding the different cycles (funding, electoral, project and publication), and incorporating continuous feedback and flow of information between communities.

**Fig. 2:**
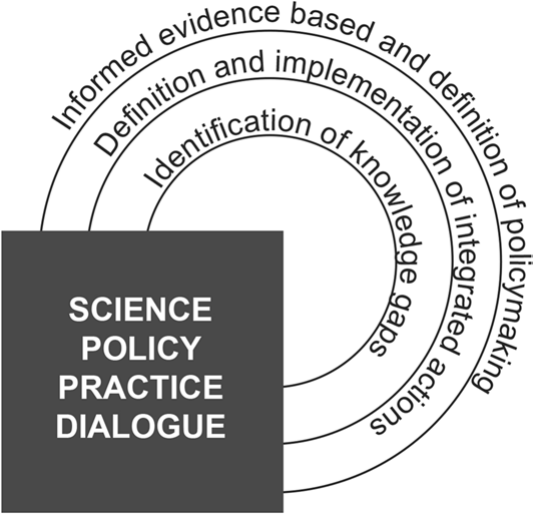
The science-policy-practice dialogue for urban resilience. Source: Authors’ elaboration

Our proposal to unlock the dialogue between science, policy and practice in resilience issue supports a circular and mutual process, as explained in Fig. 2.

Within this process, science has a double function to highlight evidence support to informed policy in decision making and to define research agenda responding to knowledge and information gaps in policy and practice. Moreover, science has the role to identify knowledge gaps in policy and practice. The dialogue among science, policy and practice is crucial to identify knowledge gaps, to define informed evidence based in policy making and to define and implement integrated actions. More in this point: (i) practice and policy academics have to implement new tools, technically supporting the knowledge of challenges and measures for vulnerability, creating a circular process and continuous learning of resilience; (ii) policy-makers have to define a local resilience agenda, supporting international and national policies; (iii) practitioners have to overcome the gaps and challenges of resilience. operationalisation, collecting and experiment a catalogue of best practices.

We firmly support that it is necessary to abandon “silo thinking” and work together to align urban resilience actions and visions with all relevant entities, fostering:

1. Mutual understanding, respect and effective communication across silos within and between communities to advance the co-production and co-generation of knowledge and empowering cities to take action.
2. City-to-city partnerships to encourage the exchange of knowledge across cities and to develop capacity.
3. Opportunities for researchers to work in municipal and local governments, and opportunities for practitioners and decision-makers to invest time in research projects.

**Fig. 3:**
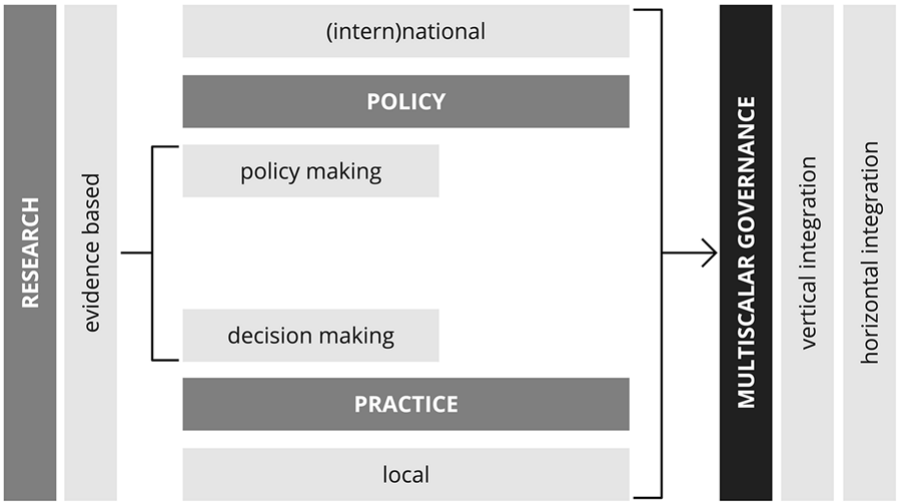
Multiscalar governance for urban resilience. Source: Authors’ elaboration

To make this process real, we need to strengthen a multiscalar governance approach characterized by a strong vertical and horizontal integration (Fig. 3).

In the first hand, vertical integration must answer to two questions: (i) how the local action contributes to achieving (int)national policy objectives (e.g. specific targets for emission reduction)?; and (ii) how national government support local action (e.g. through financial, technology and institutional capacity)? In the other hand, horizontal integration must ensure consistent cooperation between national governments ministries and local authoriti es departments competences (e.g., environment/climate, and finance).

In conclusion, this article proposes an iterative approach among science, policies and practice in implementing urban resilience, in which the three components cannot be independent. The key message is that the dialogue among science, policies and practice should not be horizontal (as commonly is, as highlighted from the Italian case study) but circular to be a tie that works. In this resilience governance approach, science has the role of helping cities experiment the best way of defining resilience, while the result of the interaction between science and practice is the starting point in setting local policies towards urban climate resilience.

## Data Availability

Not applicable.

## Footnotes

1 The IPCC Fifth Assessment Report defines adaptation as the “process of adjustment to actual or expected climate and its effects. In human systems, adaptation seeks to moderate or avoid harm or exploit beneficial opportunities”, while mitigation as “aA human intervention to reduce the sources or enhance the sinks of greenhouse gases (GHGs)”(IPCC 2014).
